# Photoactivation of Curcumin Doped Poly-Lactic-Co-Glycolic Acid Nanoparticles in Rat Model with Fixed Orthodontic Appliances

**DOI:** 10.1155/2022/3613345

**Published:** 2022-05-19

**Authors:** Sara Ghorbanpour, Maryam Pourhajibagher, Mohammad Noroozian, Hassanali Ghaffari, Abbas Bahador

**Affiliations:** ^1^Department of Microbiology, Tehran University of Medical Sciences, Tehran, Iran; ^2^Dental Research Center, Dentistry Research Institute, Tehran University of Medical Sciences, Tehran, Iran; ^3^Department of Orthodontics, School of dentistry, Ilam University of Medical Sciences, Ilam, Iran; ^4^Student Research Committee, School of dentistry, Ilam University of Medical Sciences, Ilam, Iran; ^5^Department of Orthodontics, School of Dentistry, Shahed University, Tehran, Iran; ^6^Clinical Laboratory Sciences, BioHealth Lab, Tehran, Iran

## Abstract

This study aimed to evaluate the antimicrobial effect of curcumin doped poly-lactic-co-glycolic acid nanoparticles (Cur-PLGA-Nps)-mediated antimicrobial photodynamic therapy (aPDT), as well as the probiotics on *S. mutans* in rats with fixed orthodontic appliances. Orthodontic appliances were ligated to the right maxillary of the rats. After the oral colonization of *S. mutans*, the rats were then treated in four groups including Cur-PLGA-Nps, light-emitting diode, Cur-PLGA-Nps-mediated aPDT, and probiotic (*Lactobacillus acidophilus*). After that, the *S. mutans* counts and the *gtfB* gene expression of *S. mutans* were determined on days 4, 7, 15, and 30. Probiotic and Cur-PLGA-Nps-mediated aPDT groups significantly reduced the count of *S. mutans* in a time-dependent manner (*P* < 0.05). So, probiotics and Cur-PLGA-Nps-mediated aPDT were able to reduce *S. mutans* more than other groups on the 30^th^ day. Also, there was no considerable difference between Cur-PLGA-Nps-mediated aPDT and probiotic groups in bacterial growth inhibition. The expression level of *gtfB* gene was significantly downregulated at all-time intervals after exposure to Cur-PLGA-Nps-mediated aPDT compared with untreated bacteria (*P* < 0.05). According to the results, simultaneous use of Cur-PLGA-Nps-mediated aPDT and probiotic therapeutic approaches can be suggested to increase effectiveness.

## 1. Introduction

Fixed orthodontic appliances provide convenient zones for the retention of food and cause dental plaque accumulation. Difficulty in maintaining oral hygiene in the presence of orthodontic attachments can lead to the formation of white spot lesions and dental caries. Although recent advances in the orthodontics field have enhanced the quality of treatment, however, enamel demineralization is still a common side effect accompanied with orthodontic treatment and the prevalence of white spot lesions, known as the precursor of tooth decay, in orthodontic patients has been reported to be ranged from 4.9% to 84% [1, 2].

Many techniques have been suggested to prevent or reverse enamel demineralization [3]. Topical fluoride application using fluoride varnishes and tooth pastes, casein phosphopeptide amorphous calcium phosphate, constant fluoride release from fluoride-containing elastomeric chains, glass ionomer cement, primers, and orthodontic adhesives has been proven to be beneficial, but the application of fluoride not only needs frequent visits to the clinician but also leads to discoloration of the tooth and causes reduced esthetics [4–6]. The application of antibiotics and antimicrobial mouthwashes has been adopted for suppressing the caries infection but never completely eliminates it, and these medicaments should be used at regular intervals to attain long-term results [7].

Antimicrobial photodynamic therapy (aPDT) is a relatively modern and noninvasive therapy that has been used in orthodontic patients for oral environment decontamination, eliminating periodontal pathogens, and inhibiting enamel decalcification [8]. It is based on the interaction between a light source and a photoactive compound or photosensitizer. After light radiation, photosensitizer absorbs it and transforms from ground condition to excited condition; then, it commences a series of reactions that cause generation of reactive oxygen radicals; the highly reactive oxygen species (ROS) cause devastation of the bacterial cell membrane and nucleic acid permanently leading to microbial death. Several benefits have been reported for aPDT over conventional antimicrobial agents such as rapid and broad spectrum of action, low risk to create resistant bacterial strains, minimum detrimental effect on adjacent tissue, and low risk of bacteremia [9, 10].

Curcumin (Cur) is a bioactive, organic, and phenolic substance found in turmeric and can be obtained from the rhizomes of the *Curcuma longa*. Cur has shown diverse therapeutic characteristics in medicine including antioxidant, anti-inflammatory, antitumor, and antimicrobial effects [11–13]. It has also shown considerable potential as a photosensitizer during aPDT due to its capability to absorb blue light [14–17]. Despite many therapeutic properties, the clinical application of Cur is restricted because of hydrophobicity or insolubility in water, low stability, and poor bioavailability. As a result, different drug delivery systems have been designed and assessed to overcome these limitations [18–21]. Associating Cur with different polymers to manufacture a stable nanostructured polymeric system has been suggested. The choice of carrier polymer in the oral delivery system significantly affects the pharmacodynamics and pharmacokinetics of Cur. Different materials including chitosan, cyclodextrins, and dendrimers have been used as carriers to enhance the bioavailability of Cur [22, 23]. Poly(lactic-co-glycolic acid) (PLGA) is a biodegradable copolymer that can be used as an effective carrier for drug delivery. It has been approved by U.S. Food and Drug Administration (FDA) [24, 25]. Some studies reported increased bioavailability of Cur when PLGA was used as a delivery system during oral administration [26, 27]. According to these factors, for improving the oral bioavailability of Cur, we prepared PLGA nanoparticles loaded with Cur (7% wt) and used them as a photosensitizer for aPDT.

Probiotics are bacterial cultures or live microbial nutritional supplements, which positively affect the general health of host animals by changing their gastrointestinal tract microbial balance. They act as antagonists of the pathogenic bacteria and competitively prevent the multiplication of opportunist microorganisms as they have more adhesion ability to the tissues [28]. They limit pathogens but do not restrict normal microflora. Once the pathogenic microorganisms are substituted, the recurrence of the pathogen does not happen easily. Probiotics could be available in the regular diet without causing adverse effects [28]. Several studies have found that mouthwashes and dentifrices containing probiotics have a beneficial impact on oral health by preventing halitosis, gingivitis, and dental caries [7, 29]. Studies reported the positive effect of consuming probiotic products in reducing the cariogenic bacteria levels in the saliva of orthodontic patients [30, 31].

*Streptococcu*s *mutans* is the pivotal microorganism playing a major role in the initiation and the progression of dental caries because of its great acid production ability. Also, glycosyltransferase (GTF), a streptococcal enzyme, converts glucose to glucan polymers and this viscose polysaccharide facilitates the binding of other cariogenic bacteria [32, 33]. Levels of *S. mutans*, present in dental plaque and saliva, notably are higher in patients with a high incidence of dental caries [34], and it is found in higher quantities in patients with fixed orthodontic appliances compared with nonorthodontic patients [35]. Accordingly, measuring salivary *S. mutans* count has been reported in many previous studies to be a reliable index for risk of dental caries and it has been used to determine the anticaries effect of different antimicrobial treatments [36, 37].

This study describes an aPDT approach based on a new photosensitizer to reduce the population and virulence *S. mutans* as the most important cause of dental caries in an orthodontic animal model. The novelty of this study lies in the fact that aPDT mediated by Cur-PLGA-Nps is involved in helping to reduce the population and virulence of *S. mutans* without *in vivo* adverse effects. To our knowledge, this is the first report that explores the effect of aPDT mediated by Cur-PLGA-Nps against *S. mutans* in rats with fixed orthodontic appliances. The results of this study add to the literature in highlighting the importance of the aPDT, as well as the efficacy of the provision of a new photosensitizer to reduce the population and virulence *S. mutans*, which results in the decrease of white spot lesions and dental caries during fixed orthodontic treatment. The advantages of this study include the fact that it included a new Ps, an orthodontic animal model, different treatment approaches, an examination of the changes in population and virulence *S. mutans* over time following each treatment, and a proper consideration of statistical analysis methods. So, this animal study aims to investigate the antimicrobial activity, antivirulence potency, and durability of aPDT mediated by Cur-PLGA-Nps against *S. mutans* in rats with fixed orthodontic appliances and to compare it with probiotics. The null hypothesis is that aPDT can have a more lasting antimicrobial effect than probiotics.

The remainder of the current paper is organized as follows. In sections 2.1to 2.3, we present the ethical approval code, study design, and sample size. In section 2.4, we provide the inclusion and exclusion criteria. The randomization, blinding, outcome measures, and experimental animals are described in sections 2.5to 2.8. These are followed by the experimental procedures and statistical methods in sections 2.9 and 2.10, respectively. The results of our study are shown in Section 3. In the next section of this paper, Section 4, we discuss the obtained data. In Section 5, we present the study limitations. Finally, we draw the conclusions of the current study in Section 6.

## 2. Material and Methods

### 2.1. Ethical Approval Code

This study was approved by the Ethics Committee of Tehran University of Medical Sciences (Approval code: IR.TUMS.MEDICINE.REC.1399.944) and was performed in compliance with the guideline for the care and use of laboratory animals in Iran [38].

### 2.2. Study Design and Treatment Procedure

**Figure 1:**
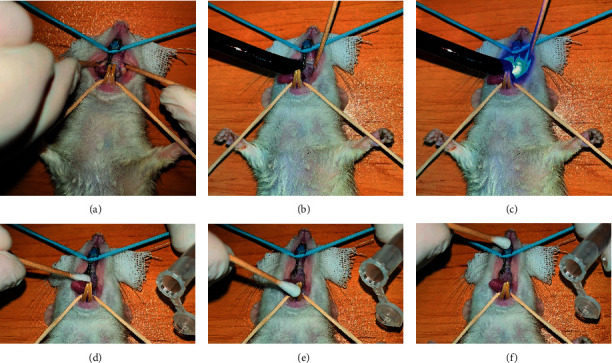
The aPDT and sampling procedures in the oral cavity of the rats with fixed orthodontic appliances: (a) Orthodontic appliance fixed in the oral cavity of the rat, (b) Before irradiation of LED during aPDT, (c) Irradiation of LED during aPDT, d-f) Sampling by a sterile swab drawn on the tooth and the contact area of the orthodontic appliance and teeth.

The animals (rats) served as their control in the present longitudinal study to minimize the uncertainty caused by the intersample differences. The experimental groups were defined as follows:

- Cur-PLGA-Nps at the concentration of 7% wt (50 mg/kg), daily in water.
- Irradiation of light-emitting diode (LED; DY400-4, Denjoy, China) at the wavelength of 450 ± 5 nm and power intensity of 1000–1400 mW/cm^2^ for 1 min.
- aPDT using Cur-PLGA-Nps and LED as described groups A and B. The aPDT procedure is shown in Figure 1.
- Probiotic *Lactobacillus acidophilus* ATCC 4356 at the concentration of 1.5 × 10^8^ colony-forming unit (CFU)/mL, daily in water.

### 2.3. Sample Size

In the current study, the resource equation approach was used to sample size calculation [39–42]. According to this approach, the acceptable range of degrees of freedom (df) for the error term in an analysis of variance (ANOVA) is between 10 and 20 [39, 41, 42]. The resource equation approach is suitable for studies whenever it is not possible to assume the effect size and the standard deviation. It also is applicable whenever the data are a quantitative variable and suitable for analysis by ANOVA [40].

Based on this approach, the number of animals per group for group comparisons using one-way ANOVA was obtained as follows:

(1) $$\begin{array}{c} n=\frac{DF}{k}+1, \end{array}$$

where *n*  is  number of animals per group, and *k*  is  number of groups according to the acceptable range of the DF (minimum [10] and maximum [20]) to obtain the minimum and maximum numbers of animals per group.

In total, the minimum and maximum numbers of animals required were as follows:

(2) $$\begin{array}{c} \text{Minimum }N=\text{Minimum }n\times k,\\ \text{Maximum }N=\text{Maximum }n\times k, \end{array}$$

where *N*  is a total number of animals.

In the current study to compare the *S. mutans* population (in CFU/mL, change = post-treatment to pretreatment) between four treatment groups (aPDT, Cur-PLGA-Nps, LED, and probiotics), the sample sizes per group were as follows:

(3) $$\begin{array}{c} \text{Minimum }n=\frac{10}{4}+1=\frac{3.5\text{ animals}}{\text{group}},\\ \text{Maximum }n=\frac{20}{4}+1=\frac{6\text{ animals}}{\text{group}}. \end{array}$$

Based on the report of Charan and Kanthariaor [41], the final sample size was adjusted for expected attrition or death of animals by the following formula:

(4) $$\begin{array}{c} \text{Corrected sample size}=\frac{\text{Sample size}}{\left(1-\left[\text{\% attrition}/100\right]\right)}. \end{array}$$

In the current study, considering 10% attrition, the final sample sizes were as follows:

(5) $$\begin{array}{c} 4\text{ Minimum animals per group}\left(\frac{3.5}{0.9}=3.8=\frac{\text{rounded up to }4\text{ animals}}{\text{group}}\right),\\ 6\text{ Maximum animals per group}\left(\frac{6}{0.9}=6.66=\frac{\text{rounded down to }6\text{ animals}}{\text{group}}\right). \end{array}$$

The total sample sizes were as follows:

(6) $$\begin{array}{c} \text{Minimum }N=\text{Minimum }n\times 4=4\times 4=16\text{ animals,}\\ \text{Maximum }N=\text{Maximum }n\times 4=6\times 4=24\text{ animals.} \end{array}$$

In conclusion, for the current study, between 4 and 6 animals per group were required. In other words, a total of 16 to 24 animals were required to keep the df within the range of 10 to 20.

### 2.4. Inclusion and Exclusion Criteria

Male Wistar rats of 10–12 weeks old, healthy, and weighing 350–380 g were included. Female Wistar rats, low weight, sick, and lazy male Wistar rats were excluded. The colonized rats with lower than 10,000 CFU/mL of *S. mutans* were also excluded.

### 2.5. Randomization

In this study, the 20 rats were randomly divided into the experimental groups with 5 samples in each group (A-D) by Random Allocation Software based on the selected type of blocking.

### 2.6. Blinding

Animals were coded before sample collection so that treatment groups could not be identified before completing the analysis. During *S. mutans* colony counting and assessment of *gtfB* mRNA expression following each treatment, a microbiologist was blinded to the treatment each animal received in which animals were grouped. During the data analysis, a person analyzing the data was blinded to the specific treatment each group received.

### 2.7. Outcome Measures

At predetermined time intervals (on days 0 [D0; as a control], 4 [D4], 7 [D7], 15 [D15], and 30 [D30]), sampling was collected using a sterile swab drawn on the tooth and the contact area of the orthodontic appliance and teeth (Figure 1). *S. mutans* colony counting and *gtfB* mRNA expression were measured as outcomes.

### 2.8. Experimental Animals

Due to minimize the potential confounders such as animal location, the Wistar rats were kept in the aseptic plastic cages, at room temperature (23 ± 2°C) with 12-hour light and dark cycle and were fed a diet including standard powdered rat food (Harlan Laboratories) and distilled water ad libitum. All animals were acclimatized to the laboratory environment for at least 1 week before the initiation of the study.

The rats were anesthetized with an intramuscular injection of a combination of 6 mg/kg xylazine hydrochloride and 50 mg/kg ketamine hydrochloride (both from Syntec, Santana de Parnaiba, Sao Paulo, Brazil) [43] before placement of orthodontic appliances. At the end of the experimental period, the animals were euthanized with an overdose of ketamine and xylazine, and sacrificed and their maxilla was resected.

### 2.9. Experimental Procedures

#### 2.9.1. Preparation of Cur-PLGA-Nps as a Photosensitizer

Cur-PLGA-NPs were synthesized using a modified single emulsion solvent method. Briefly, 60 mg of Cur and 300 mg of PLGA (both purchased from Sigma-Aldrich, Germany) were dissolved in an organic phase containing 3 mL dichloromethane and 2 mL acetone (both purchased from Merck, Germany). This mixture was emulsified in an aqueous phase containing 1% polyvinyl alcohol (30–70 kDa; Merck, Germany). This emulsion was then sonicated at 55 W for 2 min to form the final emulsion. The emulsion formed was centrifuged at 16000 rpm for 10 min to assist the removal of residual solvents. The nanoparticle pellet was then washed three times with deionized distilled water, freeze-dried, and stored at 4°C until further use.

#### 2.9.2. Cur-PLGA-Nps Characterization

Cur-PLGA-Nps were characterized for particle size, zeta potential, and polydispersity (PDI) using a MALVERN Zetasizer Ver. 6.01 (Malvern Instruments, UK) at approximately 25°C. The transmission electron microscope (TEM; Zeiss EM10 C) with an accelerating voltage of 80 Kv was used to assess the particle size and size distribution Cur-PLGA-Nps. Load and entrapment efficiency of Cur in Cur-PLGA-Nps was determined by UV-visible spectrophotometer as previously describe [44].

#### 2.9.3. Colonization of *S. mutans* in the Oral Cavity of Rats

The rat oral microbiota was removed by adding the antibiotics ampicillin and kanamycin (both 20 mg/kg) in their water container daily for 4 consecutive days and spent 3 days without antibiotic treatment. A standard strain of *S. mutans* ATCC 35668 (obtained from Iranian Biological Resource Center, Tehran, Iran) at the concentration of 1.5 × 10^8^ CFU/mL supplemented with 3% sucrose was added to the 1 g of rat food for 8 days [45]. 24 h after the last inoculation, sampling was performed to examine the establishment of bacteria. Rats that were found to have bacteria in their oral cavity were then selected for further study, and the rest of the rats underwent infection and sampling again to enter the orthodontic appliance placement phase.

#### 2.9.4. Orthodontic Appliance Placement

A closed-coil nickel-titanium spring (wire size 0.005 in., diameter 1/12 in.) (3 M Unitek, Monrovia, CA) was ligated to the right maxillary first molar by a 0.010-inch stainless steel ligature (G&H Wire Company, Franklin, IN, USA). The spring was activated to deliver a constant mesial force of 50 g to the maxillary first molar that was measured by Dontrix gauge (OSE, USA), and no reactivation was performed during the study, as previously described [46]. A notch was created between central incisors with a rotary handpiece, and then, incisors were etched with 37% phosphoric acid gel (3M, Dental products, St. Paul) for 40 s, were washed using water spray for 15 s, and then were dried using oil-free air spray for 10 s. The other side of the spring was also ligated to the ipsilateral maxillary central incisor using the same ligature wire. At last, the orthodontic appliance was fixed in place via light-cured flowable composite resin that was filled the notch and was placed around incisors to prevent the disengagement of spring. Mandibular incisors were reduced weekly to keep them out of occlusion and not cause breakage or displacement of the spring. The rats were monitored for signs of weight loss, infection, and appliance failure throughout the experiment, and in the event of these situations; animals were excluded from the study and replaced with a new one. The left side of the maxilla was considered the control for later measurements of the space formed between the first and second molars.

#### 2.9.5. Effect of Treatment on *S. Mutans* Population by Colony Counting

Following treatment and sampling, the serial dilutions were prepared, and 10 *μ*L of each dilution was cultured onto brain heart infusion (BHI) broth (Merck, Germany). The agar plates were incubated at 37°C under capnophilic conditions (5% CO_2_), and CFUs/mL was counted based on the previous study [37].

#### 2.9.6. Effect of Treatments on gtfB mRNA Expression by Quantitative Real-Time Polymerase Chain Reaction (qRT-PCR)

The super RNA extraction kit (AnaCell, Iran) was used to obtain total RNA from treated *S. mutans*. Total RNA (150 ng) was reverse transcribed in a 10 *μ*L cDNA reaction volume RevertAid First Strand cDNA Synthesis Kit (Thermo Scientific GmbH, Germany) based on the manufacturer's instructions. qRT-PCR analysis was then performed using SYBR^®^ Premix Ex Raq™ II (Tli RNaseH Plus; Takara, Korea) on Line-GeneK Real-Time PCR Detection System and Software (Bioer Technology, Hangzhou, China). The sequences of forward and reverse primers of *gtfB* and *16S rRNA* (as housekeeping) genes were as follows: *gtfB-*forward 5′-TGTTGTTACTGCTAATGAAGAA-3′ and *gtfB-*reverse 5′-GCTACTGATTGTCGTTACTG-3′; *16S rRNA-*forward 5′-GCAGAAGGGGAGAGTGGAAT -3′ and *16S rRNA-*reverse 5′-GGCCTAACACCTAGCACTCA-3′. The thermal cycling conditions were one cycle at 95°C for 5 min followed by 40 cycles at 95°C for 10 s, 54°C for 10 s, and 72°C for 30 s.

### 2.10. Statistical Methods

The obtained data were analyzed through SPSS version 26 software. To avoid possible error in statistical analyses due to low sample size (n = 5 per group), the sample distribution was considered as non-normal and the Friedman test was used to compare the mean of CFU/mL of *S. mutans* in the four treatment groups through the five subsequent measurement time points as a nonparametric test. The Wilcoxon test was applied for pairwise comparisons. To compare the treatment groups with each other at each of the intervals, the Kruskal–Wallis test was used after the normalization of data. The significant Kruskal–Wallis tests were followed by the Mann–Whitney post hoc test. Since one microbial test was done following each treatment, in the correction of the alpha level using the Bonferroni method, the original value (0.05) does not change (i.e. 0.05/1), and a value less than 0.05 was considered significant. The raw cycle threshold (Ct) values of *gtfB* qRT-PCR analysis following each treatment were normalized to the housekeeper gene (*16S rRNA*) using an analysis of covariance method, before relative fold change over D0 (baseline) was calculated using Livak and Schmittgen (2^−ΔΔCT^) method [47].

## 3. Results

### 3.1. Confirmation of Cur-PLGA-Nps Synthesis

**Figure 2:**
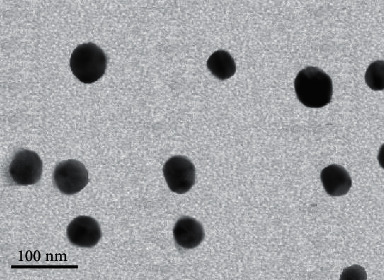
Transmission electron microscope micrographs of Cur-PLGA-Nps, (scale bar represents 100 nm).

The surface morphology of the nanoparticles encapsulating Cur, prepared by emulsion technique, was determined by TEM. The spherical and smooth structures of Cur-PLGA-Nps with a more or less uniform size distribution are shown in Figure 2. The size, zeta potential, and PDI of Cur-PLGA-Nps were 63 ± 2.1 nm, −15.1 ± 1.3 Mv, and 0.28, respectively, as measured by MALVERN Zetasizer. According to the results, the percentage of load and entrapment efficiency of Cur in Cur-PLGA-Nps was 64.5 ± 4.05% and 25.7 ± 0.18%, respectively.

### 3.2. Antimicrobial Effect of Treatments against *S. mutans*

The obtained and normalized data of *S. mutans* following treatment with different groups at the different time intervals are given in supplemental data (S-Tables 1 and 2). The minimum, maximum, mean, median, standard deviation, and 95% confidence interval of S*. mutans* CFU/mL following each treatment in different time points are shown in Table 1. Additionally, as the results show, the antimicrobial properties of aPDT and probiotic are time-dependent, so the highest reduction in the number of *S. mutans* was observed on Day 30 of the study.

As shown in Table 2, the Friedman test revealed significant differences between the mean of CFU/mL of *S. mutans* in each treatment group through the five subsequent measurement time points except LED group. The Wilcoxon test revealed that the mean of CFU/mL of *S. mutans* in aPDT group differed statistically significantly between time points, but no significant differences were found following LED treatment between time points (*P* > 0.05; Table 3). There was also a statistically significant difference in *S. mutans* population following Cur-PLGA-Nps treatment only between D0 with others (*P* > 0.05; Table 3) and following probiotic treatment between D0 with others, D4 with D7, and D4 with D30 (all *P* > 0.05; Table 3).

Kruskal–Wallis test revealed significant differences of CFU/mL of *S. mutans* between treatment groups with each other at each of the intervals except D0 (Table 4). Post hoc Mann–Whitney pairwise comparison following significant Kruskal–Wallis tests indicated significant differences in all treatment groups at each of the intervals except for D0 in all treatment groups and also between the aPDT and probiotic treatment groups at D4 (Table 5).

### 3.3. Monitoring of gtfB Gene Expression of Treated *S. mutans*

**Figure 3:**
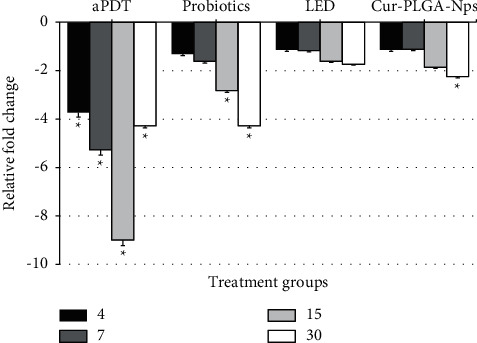
Changes in gene expression of *gtfB* following treatment with different groups at the different time intervals. The error bars represent the standard deviation. ^*∗*^Significant differences according to the control group (day 0), *P* < 0.05.

Changes in gene expression of *gtfB* at the different time intervals following treatment with various treatment groups are shown in Figure 3. The expression level of *gtfB* gene significantly decreased at all-time intervals after exposure to aPDT compared to untreated *S. mutans* isolates (*P* < 0.05). Probiotics on days 15 and 30 and Cur-PLGA-Nps on Day 30 were able to downregulate the gene expression levels by 2.82-, 4.28-, and 2.25-fold, respectively (*P* < 0.05), while LED could not significantly reduce gene expression of *gtfB* within a month (*P* > 0.05).

## 4. Discussion

The level of patient care and the quality of treatments have been raised by recent advances in orthodontics. Many people want to receive orthodontic treatment as a result of the growing population, higher patient awareness, and changing lifestyles; however, increased risk of enamel decalcification is still an unsolved and frequent problem associated with fixed orthodontic treatments [48]. The aPDT has successfully been used in orthodontic patients for oral cavity decontamination and reducing the count of cariogenic bacteria [49], but it has not been able to prevent the regrowth of remaining pathogens or reinfection and this means that it should be repeated at regular intervals to achieve long-term results, especially in orthodontic patients with relatively long treatment time [50]. The application of probiotics may have more long-lasting antimicrobial effects during orthodontic treatment as these microbial food supplements have a higher affinity to tissues in comparison with cariogenic microorganisms and will delay the reintroduction of the pathogens [51]; therefore, in this study, the antimicrobial effects of aPDT and systemic use of probiotics were assessed and compared with each other in rats with fixed orthodontic appliances simulating patients with fixed orthodontic appliances.

In the study by Panhoca et al. [52], aPDT mediated with Cur associated with surfactant decreased level of *S. mutans* in the saliva of orthodontic patients remarkably and its efficacy was same as 0.12% chlorhexidine gluconate, the gold standard antimicrobial mouth rinse. In their study, aPDT was done for one session and its antimicrobial effect was assessed immediately without any follow-up period. They reported a higher reduction in the count of *S. mutans* after aPDT combined with surfactant compared to aPDT alone. In our study, Cur combined with a different carrier polymer (PLGA) was used. According to the finding, the antimicrobial activity of aPDT is time-dependent, so the highest reduction in *S. mutans* count was observed on Day 30. Also, aPDT compared with Cur-PLGA-Nps and LED alone was able to significantly inhibit bacterial growth.

Gomez et al. [53] compared methylene blue-mediated aPDT with ultrasonic scaling (US) as two different prophylactic approaches in orthodontic patients; these two methods showed similar efficacy in reducing levels of salivary *S. mutans* until 3 months of follow-up; however, the levels of salivary *S. mutans* increased again in 6-month and 9-month follow-ups; these findings point to need for repetition of both aPDT and the US to achieve long-term results. Paschoal et al. [54] investigated the longitudinal impact of Cur-mediated aPDT on the gingival bleeding index (GBI) and plaque index (PI) in adolescents during fixed orthodontic treatment. In their study, aPDT was effectively same as chlorhexidine varnish 2% in decreasing GBI after 1-month and 3-month follow-ups. Also, they did not observe a difference in PI between the group that received varnish and the group that received aPDT after 1-month follow-up, but PI was significantly higher in the aPDT group in comparison with the varnish group after 3-month follow-up. A nonsubstantial impact of aPDT after a 3-month follow-up on PI as an indicator for dental plaque colonization could be explained according to the formation of biofilm structures.

Dental biofilm is known for high pathogenicity and low vulnerability to aPDT and environmental assaults as the presence of biofilm matrix causes structural integrity and inhibits the penetration of antimicrobial agents [55]. By contrast, in our study, aPDT mediated with Cur-PLGA-Nps showed increasing antimicrobial effect during 1-month follow-up; this inconsistency may be due to shorter follow-up time and assessment of different parameters, which was the count of salivary *S. mutans* in the planktonic form. In line with previous studies, our study showed that aPDT mediated with Cur-PLGA-Nps is an effective alternative for reducing levels of salivary *S. mutans* during fixed orthodontic treatment; however, recolonization of pathogens has remained an unsolved issue, and repeating aPDT to attain long-term results is crucial same as the conventional antimicrobial treatments. In addition, dental plaque consisting of diverse microorganisms that are surrounded by a matrix of polymers seems to be more persistent to aPDT, so applying methods that prevent pathogens colonization and interaction to create more harmful complexes such as probiotics may be a more practical approach during orthodontic treatments.

Jose et al. [56] investigated and compared the effect of a short-term systemic consumption of probiotics and topical application of probiotics in the form of toothpaste on the count of *S. mutans* in plaque around fixed orthodontic appliances. Plaque samples were collected from the labial surface of orthodontic brackets before the intervention and after 30 days of continuous use of probiotics, and the presence of *S. mutans* was assessed using RT-PCR. They observed a significant reduction in the levels of *S. mutans* in the group that used probiotics systemically and the group that use it topically, both. Alp et al. [57] assessed the effect of regular systemic and topical consumption of probiotic products on the count of *S. mutans* and *Lactobacillus* in the saliva of orthodontic patients. Three groups with 15 individuals in each including control, kefir group in which patients consumed kefir (Ataturk Orman Ciftligi, Ankara, Turkey) drink twice a day, and probiotic toothpaste group in which participants brush their teeth with the probiotic product (GD toothpaste; Dental Asia Manufacturing, Shah Alam, Selangor, Malaysia) twice a day. Saliva samples were collected at beginning of the study, after 3 weeks, and after 6 weeks to evaluate its flow rate, buffer capacity, *S. mutans*, and *Lactobacillus* levels. They reported that regular systemic or topical use of probiotics during fixed orthodontic treatment significantly decreases the count of *S. mutans* and *Lactobacillus* in the saliva of orthodontic patients. In a systematic review of randomized clinical trials by Pietri et al. [58], the effect of probiotics on oral health maintenance during orthodontic treatment was assessed. Eight of 9 included studies indicated that probiotic therapy enhances oral health in orthodontic patients, and 7 of 9 studies reported that probiotic therapy decreases the count of pathogenic bacteria in the oral biofilm or/and saliva. Shah et al. [59] evaluated the antimicrobial efficacy of probiotics and compared it with chlorhexidine, the gold standard antimicrobial mouthwash, in individuals undergoing fixed orthodontic treatment. 30 healthy participants undergoing fixed orthodontic treatment were randomly distributed into 3 groups including 0.2% chlorhexidine mouthwash, probiotic mouthwash, and control. Saliva samples were collected at the start of the study and once every week for 4 weeks and then were spread over mitis salivarius-bacitracin culture medium, and CFU/mL was determined. They observed that probiotics are as effective as chlorhexidine in decreasing levels of salivary *S. mutans*. The results of our study were consistent with previous studies and showed a significant reduction in the salivary count of *S. mutans* after systemic application of probiotics in rats during 30 days of follow-up. An interesting finding in our study was that the comparative intergroup statistical analysis showed similar bactericidal efficacy of aPDT and systemic probiotic therapy in reducing levels of salivary *S. mutans*.

In the current study, the antivirulence activity of aPDT using Cur-PLGA-Nps plus LED was evaluated. The results showed that the expression level of *gtfB* gene significantly decreased at all-time intervals after exposure to aPDT compared with the control group (untreated *S. mutans* isolates), while probiotics were able to reduce gene expression 2.82- and 4.28-fold on days 15 and 30, respectively. In addition, significant differences in downregulation of gene expression were also observed between aPDT and other groups.

## 5. Study Limitations

The main limitation of the present study was the small sample size in all groups. The smaller sample size is because many rats exposed to S. mutans did not colonize (at least 10,000 CFU/mL) and did not meet the current inclusion criteria. This was one of the difficulties we faced during this animal model of fixed orthodontics study. This may be due to the lack of sufficient bacterial receptors on the enamel surfaces of rats exposed to *S. mutans*.

Other limitations of the current study were as follows: (1) no effect size was used in the calculation of the sample size. (2) Duration of the study (30 days). The average orthodontic treatment encompasses two years, but the current study was limited to one-month period based on orthodontic recall appointments during fixed treatment. A one-month time span does not allow for full implementation of a new approach for controlling dental caries. Long-term studies are required to establish the true effects of aPDT mediated by Cur-PLGA-Nps against *S. mutans* in rats with fixed orthodontic appliances. (3) Measuring one bacterial strain (S. mutans). Dental caries is recognized as an infectious disease caused by multispecies acid-producing bacteria. Thus, further studies with multispecies cariogenic bacteria are necessary to better understand the anticariogenic effects of aPDT mediated by Cur-PLGA-Nps.

## 6. Conclusion

aPDT and systemic probiotic therapy showed similar efficacy in reducing the level of *S. mutans* in an animal model with fixed orthodontic appliances. To increase effectiveness, the simultaneous use of probiotics and aPDT is recommended. However, more clinical studies with longer follow-up times are required.

## Figures and Tables

**Table 1:** The minimum, maximum, mean, median, standard deviation, and 95% confidence interval of *S. mutans* CFU/mL following each treatment in different time intervals.

Groups	Time intervals	Minimum	Maximum	Mean	Median	Standard deviation	95% confidence interval	
							Lower bound	Upper bound
Cur-PLGA-nps	D0	9.60 *E* + 7	1.45 *E* + 8	116000000	103000000	23979157.61656	86225943.2803	145774056.7197
	D4	4.30 *E* + 7	9.90 *E* + 7	68000000	62000000	23452078.79912	38880398.0721	97119601.9279
	D7	4.40 *E* + 7	7.50 *E* + 7	59000000	57000000	12227019.26064	43818150.3787	74181849.6213
	D15	3.80 *E* + 7	8.40 *E* + 7	57000000	50000000	20124611.79750	32011994.0532	81988005.9468
	D30	2.80 *E* + 7	8.20 *E* + 7	52000000	43000000	24093567.60631	22083884.5150	81916115.4850

Probiotic	D0	1.14 *E* + 8	2.21 *E* + 8	163000000	159000000	41527099.58569	111437295.4946	214562704.5054
	D4	1.30 *E* + 6	7.10 *E* + 6	4200000	4800000	2277059.50735	1372657.1880	7027342.8120
	D7	1.20 *E* + 6	6.30 *E* + 6	3200000	2400000	2221485.98915	441660.8248	5958339.1752
	D15	1.30 *E* + 6	2.80 *E* + 6	2100000	2400000	659545.29791	1281066.3484	2918933.6516
	D30	1.10 *E* + 6	3.30 *E* + 6	1920000	1400000	944457.51625	747301.1042	3092698.8958

LED	D0	9.10 *E* + 7	1.74 *E* + 8	124000000	121000000	32434549.48045	83727187.6122	164272812.3878
	D4	6.80 *E* + 7	1.72 *E* + 8	123600000	114000000	41890333.01372	71586281.6241	175613718.3759
	D7	8.40 *E* + 7	1.57 *E* + 8	122000000	111000000	31048349.39252	83448382.3557	160551617.6443
	D15	8.30 *E* + 7	1.57 *E* + 8	114600000	102000000	32623611.08155	74092436.6286	155107563.3714
	D30	7.40 *E* + 7	1.61 *E* + 8	115800000	123000000	36779070.13506	70132752.7259	161467247.2741

aPDT	D0	1.07 *E* + 8	1.93 *E* + 8	155800000	156000000	34171625.65638	113370322.6624	198229677.3376
	D4	3.06 *E* + 6	5.10 *E* + 6	3850000	3520000	832826.51255	2815909.3026	4884090.6974
	D7	840000.00	960000	902000	920000	50199.60159	839668.9620	964331.0380
	D15	42000.00	68000	52800	53000	10521.40675	39735.9480	65864.0520
	D30	1100.00	4600	2580	2700	1409.60988	829.7382	4330.2618

D0: as a control, D4: fourth day, D7: seventh day, D15: fifteenth day, and D30: thirtieth day.

**Table 2:** Results from comparisons of CFU/mL of *S. mutans* between the four treatment groups through the five subsequent measurement time points based on Friedman test.

	Treatment group			
	aPDT	Probiotic	Cur-PLGA-Nps	LED
Chi-square	20.00	15.52	10.88	2.06
df	4	4	4	4
Asymp. Sig	.00	.00	.028	.725

**Table 3:** Results from pairwise comparisons of CFU/mL of *S. mutans* between the time points with each other, within each of the treatment groups separately based on the Wilcoxon signed ranks test.

	Treatment group	D0-D4	D0-D7	D0-D15	D0-D30	D4-D7	D4-D15	D4-D30	D7-D15	D7-D30	D15-D30
Asymp. Sig. (2-Tailed)	aPDT	.043	.043	.043	.043	.043	.043	.043	.043	.043	.043
	Probiotic	.043	.043	.043	.043	.043	.104	.043	.225	.225	.588
	Cur-PLGA-nps	.043	.043	.043	.043	.892	.343	.225	.893	.500	.500
	LED	.686	.892	.588	.686	.686	.500	.893	.223	.500	.715

D0: as a control, D4: fourth day, D7: seventh day, D15: fifteenth day, and D30: thirtieth day.

**Table 4:** Results from comparisons of CFU/mL of *S. mutans* between treatment groups with each other at each of the intervals, based on the Kruskal-Wallis test.

	Time intervals				
	D0	D4	D7	D15	D30
Kruskal-wallis	6.28	15.57	17.85	17.60	17.85
df	3	3	3	3	3
Asymp. Sig	.099	.001	.000	.001	.000

D0: as a control, D4: fourth day, D7: seventh day, D15: fifteenth day, and D30: thirtieth day.

**Table 5:** Results from pairwise comparisons of CFU/mL of *S. mutans* between treatment groups with each other at each of the intervals, based on the Mann–Whitney test.

	Pairwise comparisons between	D0	D4	D7	D15	D30
Asymp. Sig. (2-Tailed)	aPDT-probiotic	.754	.754	.009	.009	.005
	aPDT-cur-PLGA-nps	.067	.009	.009	.009	.005
	aPDT-LED	.117	.009	.009	.009	.005
	Probiotic-cur-PLGA-nps	.076	.009	.009	.009	.009
	Probiotic-LED	.117	.009	.009	.009	.009
	Cur-PLGA-nps-LED	.917	.028	.009	.016	.016

D0: as a control, D4: fourth day, D7: seventh day, D15: fifteenth day, and D30: thirtieth day.

## Data Availability

All datasets supporting the conclusions of this article are included within the article.

## Supplementary Materials

Supplementary Materials The raw data obtained of *S. mutans* CFU/mL following treatment with Cur-PLGA-Nps, probiotic, LED, and aPDT groups at the time intervals of days 0 [D0; as a control], 4 [D4], 7 [D7], 15 [D15], and 30 [D30] are represented in Table S1. Also, the normalized data of *S. mutans* following Cur-PLGA-Nps, probiotic, LED, and aPDT groups at different time intervals (D0, D4, D7, D15, and D30) are shown in Table S2. The comparison of the treatment groups with each other was done after the normalization of data.
