# Supplementary material for: Photoactivation of Curcumin Doped Poly-Lactic-Co-Glycolic Acid Nanoparticles in Rat Model with Fixed Orthodontic Appliances
Source: ScientificWorldJournal. 2022 May 19;2022:3613345. doi: 10.1155/2022/3613345 (PMC9135542; doi:10.1155/2022/3613345)
Supplement: Supplementary Materials — The raw data obtained of S. mutans CFU/mL following treatment with Cur-PLGA-Nps, probiotic, LED, and aPDT groups at the time intervals of days 0 [D0; as a control], 4 [D4], 7 [D7], 15 [D15], and 30 [D30] are represented in Table S1. Also, the normalized data of S. mutans following Cur-PLGA-Nps, probiotic, LED, and aPDT groups at different time intervals (D0, D4, D7, D15, and D30) are shown in Table S2. The comparison of the treatment groups with each other was done after the normalization of data. [file 3613345.f1.zip › 3613345.f1/Author Checklist - Full.pdf]

NOTE: Please save this file locally before filling in the table, DO NOT work on the file within your internet browser as changes will not be saved. Adobe Acrobat Reader (available free here) is recommended for completion.

ARRIVE

The ARRIVE guidelines 2.0: author checklist

The ARRIVE Essential 10

These items are the basic minimum to include in a manuscript. Without this information, readers and reviewers cannot assess the reliability of the findings.

Item		Recommendation	Section/line number, or reason for not reporting
**Study design**	1	For each experiment, provide brief details of study design including:	
		a. The groups being compared, including control groups. If no control group has been used, the rationale should be stated.	Material and methods, sub heading "2.2. Study design and treatment procedure". lines 2-9.
		b. The experimental unit (e.g. a single animal, litter, or cage of animals).	Material and methods, sub heading "2.2. Study design and treatment procedure". line 1.
**Sample size**	2	a. Specify the exact number of experimental units allocated to each group, and the total number in each experiment. Also indicate the total number of animals used.	Material and methods, sub [illegible]
		b. Explain how the sample size was decided. Provide details of any *a priori* sample size calculation, if done.	Material and methods, sub [illegible]
**Inclusion and exclusion criteria**	3	a. Describe any criteria used for including and excluding animals (or experimental units) during the experiment, and data points during the analysis. Specify if these criteria were established *a priori*. If no criteria were set, state this explicitly.	Material and methods, sub [illegible]
		b. For each experimental group, report any animals, experimental units or data points not included in the analysis and explain why. If there were no exclusions, state so.	In the current study, there were no
		c. For each analysis, report the exact value of *n* in each experimental group.	Material and methods, sub
**Randomisation**	4	a. State whether randomisation was used to allocate experimental units to control and treatment groups. If done, provide the method used to generate the randomisation sequence.	Material and methods, sub heading "2.5.
		b. Describe the strategy used to minimise potential confounders such as the order of treatments and measurements, or animal/cage location. If confounders were not controlled, state this explicitly.	Material and methods, sub heading "2.8.
**Blinding**	5	Describe who was aware of the group allocation at the different stages of the experiment (during the allocation, the conduct of the experiment, the outcome assessment, and the data analysis).	Material and methods, sub heading "2.6. [illegible]
**Outcome measures**	6	a. Clearly define all outcome measures assessed (e.g. cell death, molecular markers, or behavioural changes).	Material and methods, sub [illegible]
		b. For hypothesis-testing studies, specify the primary outcome measure, i.e. the outcome measure that was used to determine the sample size.	Not applicable.
**Statistical methods**	7	a. Provide details of the statistical methods used for each analysis, including software used.	methods, sub heading "2.10. [illegible]
		b. Describe any methods used to assess whether the data met the assumptions of the statistical approach, and what was done if the assumptions were not met.	methods, sub heading "2.10. [illegible]
**Experimental animals**	8	a. Provide species-appropriate details of the animals used, including species, strain and substrain, sex, age or developmental stage, and, if relevant, weight.	Material and methods, sub [illegible]
		b. Provide further relevant information on the provenance of animals, health/immune status, genetic modification status, genotype, and any previous procedures.	Material and methods, sub [illegible]
**Experimental procedures**	9	For each experimental group, including controls, describe the procedures in enough detail to allow others to replicate them, including:	Material and methods, sub
		a. What was done, how it was done and what was used.	Material and methods, sub
		b. When and how often.	Material and methods, sub
		c. Where (including detail of any acclimatisation periods).	Material and methods, sub
		d. Why (provide rationale for procedures).	
**Results**	10	For each experiment conducted, including independent replications, report:	
		a. Summary/descriptive statistics for each experimental group, with a measure of variability where applicable (e.g. mean and SD, or median and range).	Results section, sub heading 3.1. to 3.3.
		b. If applicable, the effect size with a confidence interval.	Results section, sub heading 3.1. to 3.3.

The Recommended Set

These items complement the Essential 10 and add important context to the study. Reporting the items in both sets represents best practice.

Item		Recommendation	Section/line number, or reason for not reporting
**Abstract**	11	Provide an accurate summary of the research objectives, animal species, strain and sex, key methods, principal findings, and study conclusions.	Abstract, lines 1-21.
**Background**	12	a. Include sufficient scientific background to understand the rationale and context for the study, and explain the experimental approach.	Introduction, para 1-7.
		b. Explain how the animal species and model used address the scientific objectives and, where appropriate, the relevance to human biology.	Introduction, para 1-7.
**Objectives**	13	Clearly describe the research question, research objectives and, where appropriate, specific hypotheses being tested.	Introduction, para 7, lines 1-15.
**Ethical statement**	14	Provide the name of the ethical review committee or equivalent that has approved the use of animals in this study, and any relevant licence or protocol numbers (if applicable). If ethical approval was not sought or granted, provide a justification.	Material and methods, sub heading "2.1. Ethical approval code" lines 1-3.
**Housing and husbandry**	15	Provide details of housing and husbandry conditions, including any environmental enrichment.	Material and methods, sub heading, 2.8. Exper
**Animal care and monitoring**	16	a. Describe any interventions or steps taken in the experimental protocols to reduce pain, suffering and distress.	Material and methods, sub heading "2.8. Experimental animals" para 2, lines 1-7.
		b. Report any expected or unexpected adverse events.	unexpected adverse not found.
		c. Describe the humane endpoints established for the study, the signs that were monitored and the frequency of monitoring. If the study did not have humane endpoints, state this.	The study did not have humane endpoints.
**Interpretation/ scientific implications**	17	a. Interpret the results, taking into account the study objectives and hypotheses, current theory and other relevant studies in the literature.	Discussion section, para 1-5.
		b. Comment on the study limitations including potential sources of bias, limitations of the animal model, and imprecision associated with the results.	Discussion section, para 7.
**Generalisability/ translation**	18	Comment on whether, and how, the findings of this study are likely to generalise to other species or experimental conditions, including any relevance to human biology (where appropriate).	Discussion section, para 1.
**Protocol registration**	19	Provide a statement indicating whether a protocol (including the research question, key design features, and analysis plan) was prepared before the study, and if and where this protocol was registered.	Material and methods, sub heading "2.,.2, 2.9.3., 2.9.4, and 2.9.5.
**Data access**	20	Provide a statement describing if and where study data are available.	All obtained data such as row data are includec
**Declaration of interests**	21	a. Declare any potential conflicts of interest, including financial and non-financial. If none exist, this should be stated.	Conflict of interest<br>The authors declare that there is no conflict of interests regarding the publication of this paper.
		b. List all funding sources (including grant identifier) and the role of the funder(s) in the design, analysis and reporting of the study.	This research was supported by the Tehran University of Medical Sciences and Health Services (Grant No. 98-3-101-45415).
