# Supplementary material for: Photoactivation of Curcumin Doped Poly-Lactic-Co-Glycolic Acid Nanoparticles in Rat Model with Fixed Orthodontic Appliances
Source: ScientificWorldJournal. 2022 May 19;2022:3613345. doi: 10.1155/2022/3613345 (PMC9135542; doi:10.1155/2022/3613345)
Supplement: Supplementary Materials — The raw data obtained of S. mutans CFU/mL following treatment with Cur-PLGA-Nps, probiotic, LED, and aPDT groups at the time intervals of days 0 [D0; as a control], 4 [D4], 7 [D7], 15 [D15], and 30 [D30] are represented in Table S1. Also, the normalized data of S. mutans following Cur-PLGA-Nps, probiotic, LED, and aPDT groups at different time intervals (D0, D4, D7, D15, and D30) are shown in Table S2. The comparison of the treatment groups with each other was done after the normalization of data. [file 3613345.f1.zip › 3613345.f1/Supplemental tables-R2.docx]

**Table S1.** The obtained data of *S. mutans* following treatment with different groups at the different time intervals.

| Day=30 | Day=15 | Day=7 | Day=4 | Day=0 | Groups |
| --- | --- | --- | --- | --- | --- |
| 4.3×10^7^ | 8.4×10^7^ | 7.5×10^7^ | 8.5×10^7^ | 14.5×10^7^ | **Cur-PLGA-Nps** |
| 2.8×10^7^ | 7.2×10^7^ | 6.2×10^7^ | 6.2×10^7^ | 13.9×10^7^ |  |
| 7.3×10^7^ | 3.8×10^7^ | 5.7×10^7^ | 4.3×10^7^ | 9.7×10^7^ |  |
| 3.4×10^7^ | 4.1×10^7^ | 4.4×10^7^ | 9.9×10^7^ | 10.3×10^7^ |  |
| 2.2×10^7^ | 5.0×10^7^ | 6.7×10^7^ | 5.1×10^7^ | 9.6×10^7^ |  |
|  | | | | | |
| 1.1×10^6^ | 2.4×10^6^ | 1.2×10^6^ | 1.3×10^6^ | 18.4×10^7^ | **Probiotic** |
| 2.5×10^6^ | 1.3×10^6^ | 1.4×10^6^ | 4.8×10^6^ | 13.7×10^7^ |  |
| 1.4×10^6^ | 1.5×10^6^ | 2.4×10^6^ | 2.6×10^6^ | 22.1×10^7^ |  |
| 1.3×10^6^ | 2.5×10^6^ | 6.3×10^6^ | 7.1×10^6^ | 15.9×10^7^ |  |
| 3.3×10^6^ | 2.8×10^6^ | 4.7×10^6^ | 5.2×10^6^ | 11.4×10^7^ |  |
|  | | | | | |
| 12.3×10^7^ | 14.1×10^7^ | 15.7×10^7^ | 17.2×10^7^ | 17.4×10^7^ | **LED** |
| 7.4×10^7^ | 9.0×10^7^ | 8.45×10^7^ | 11.4×10^7^ | 10.1×10^7^ |  |
| 8.3×10^7^ | 8.3×10^7^ | 10.7×10^7^ | 15.8×10^7^ | 13.3×10^7^ |  |
| 16.1×10^7^ | 10.2×10^7^ | 11.1×10^7^ | 10.6×10^7^ | 9.1×10^7^ |  |
| 13.8×10^7^ | 15.7×10^7^ | 15.1×10^7^ | 6.8×10^7^ | 12.1×10^7^ |  |
|  | | | | | |
| 1.1×10^3^ | 3.7×10^4^ | 9.3×10^5^ | 7.3×10^6^ | 18.2×10^7^ | **aPDT** |
| 1.4×10^3^ | 2.4×10^4^ | 12.4×10^5^ | 15.2×10^6^ | 15.6×10^7^ |  |
| 4.6×10^3^ | 6.8×10^4^ | 9.6×10^5^ | 10.6×10^6^ | 19.3×10^7^ |  |
| 2.7×10^3^ | 4.2×10^4^ | 8.6×10^5^ | 12.7×10^6^ | 10.7×10^7^ |  |
| 3.1×10^3^ | 9.3×10^4^ | 5.2×10^5^ | 8.1×10^6^ | 14.1×10^7^ |  |

**Abbreviation:** D0: as a control, D4: fourth day, D7: seventh day, D15: fifteenth day, and D30: thirtieth day.

**Table S2.** The normalized data of *S. mutans* following treatment with different groups at the different time intervals.

| Day=30 | Day=15 | Day=7 | Day=4 | Day=0 | Groups |
| --- | --- | --- | --- | --- | --- |
| .00 | .02 | .42 | 1.40 | 82.35 | **aPDT** |
| .00 | .01 | .37 | 1.59 | 70.58 |  |
| .00 | .03 | .43 | 1.38 | 87.30 |  |
| .00 | .01 | .38 | 1.93 | 48.41 |  |
| .00 | .02 | .41 | 2.30 | 63.80 |  |
|  | | | | | |
| .49 | 1.08 | .54 | .58 | 83.25 | **Probiotic** |
| 1.13 | .58 | .63 | 2.17 | 61.99 |  |
| .63 | .67 | 1.10 | 1.16 | 100.00 |  |
| .58 | 1.13 | 2.85 | 3.21 | 71.94 |  |
| 1.49 | 1.26 | 2.12 | 2.35 | 51.58 |  |
|  | | | | | |
| 19.45 | 38.00 | 33.93 | 38.46 | 65.61 | **Cur-PLGA-Nps** |
| 12.66 | 32.57 | 23.52 | 28.05 | 62.89 |  |
| 33.03 | 17.19 | 25.79 | 19.45 | 43.89 |  |
| 15.38 | 18.55 | 19.90 | 44.79 | 46.60 |  |
| 37.10 | 22.62 | 30.31 | 23.07 | 43.43 |  |
|  | | | | | |
| 55.56 | 63.80 | 71.04 | 77.82 | 78.73 | **LED** |
| 33.48 | 40.72 | 38.00 | 51.58 | 45.70 |  |
| 37.55 | 37.55 | 48.41 | 71.49 | 60.18 |  |
| 72.85 | 46.15 | 50.22 | 47.96 | 41.17 |  |
| 62.44 | 71.04 | 68.32 | 30.76 | 54.75 |  |

**Abbreviation:** D0: as a control, D4: fourth day, D7: seventh day, D15: fifteenth day, and D30: thirtieth day.
